# SegCorr a statistical procedure for the detection of genomic regions of correlated expression

**DOI:** 10.1186/s12859-017-1742-5

**Published:** 2017-07-11

**Authors:** Eleni Ioanna Delatola, Emilie Lebarbier, Tristan Mary-Huard, François Radvanyi, Stéphane Robin, Jennifer Wong

**Affiliations:** 1 AgroParisTech UMR518, Paris, 75005 France; 2 INRA UMR518, Paris, 75005 France; 3 Institut Curie, PSL Research University, Cedex 05, Paris, 75248 France; 4 CNRS UMR144, Equipe Labellisee par La Ligue Nationale contre le Cancer, Cedex 05, Paris, 75248 France; 5 INRA, UMR 0320 - UMR 8120 Genetique Quantitative et Evolution-Le Moulon, Gif-sur-Yvette, F-91190 France; 6 Molecular Oncology Unit, Department of Biochemistry, Hospital Saint Louis, AP-HP, Cedex 10, Paris, 75475 France; 7 Université Paris Diderot, Sorbonne Paris Cité, CNRS UMR7212/INSERM U944, Cedex 10, Paris, 75475 France

**Keywords:** Gene expression, Chromosomes, Correlation matrix segmentation, CNV, DNA Methylation, SegCorr

## Abstract

**Background:**

Detecting local correlations in expression between neighboring genes along the genome has proved to be an effective strategy to identify possible causes of transcriptional deregulation in cancer. It has been successfully used to illustrate the role of mechanisms such as copy number variation (CNV) or epigenetic alterations as factors that may significantly alter expression in large chromosomal regions (gene silencing or gene activation).

**Results:**

The identification of correlated regions requires segmenting the gene expression correlation matrix into regions of homogeneously correlated genes and assessing whether the observed local correlation is significantly higher than the background chromosomal correlation. A unified statistical framework is proposed to achieve these two tasks, where optimal segmentation is efficiently performed using dynamic programming algorithm, and detection of highly correlated regions is then achieved using an exact test procedure. We also propose a simple and efficient procedure to correct the expression signal for mechanisms already known to impact expression correlation. The performance and robustness of the proposed procedure, called SegCorr, are evaluated on simulated data. The procedure is illustrated on cancer data, where the signal is corrected for correlations caused by copy number variation. It permitted the detection of regions with high correlations linked to epigenetic marks like DNA methylation.

**Conclusions:**

SegCorr is a novel method that performs correlation matrix segmentation and applies a test procedure in order to detect highly correlated regions in gene expression.

**Electronic supplementary material:**

The online version of this article (doi:10.1186/s12859-017-1742-5) contains supplementary material, which is available to authorized users.

## Background

In the last decade, the study of local co-expression of neighboring genes along the chromosome has become a question of major importance in cancer biology [6]. The development of “Omics” technologies have permitted the identification of several mechanisms inducing local gene regulation, that may be due to a common transcription factor [11] or common epigenetic marks [14, 34]. Copy number variation due to polymorphism or to genomic instability in cancer is also a possible cause for observing a correlation between neighboring genes [1], as their expressions are likely to be affected by the same copy number variation (CNV). It has further been observed that local regulations may occur in specific nuclear domains, as the nuclear region is an environment which may favor or not transcription [4].

Investigating the impact of a specific source of regulation (TF, CNV, epigenetic modifications such as DNA methylation and histone modifications) on the expression has now become a common practice for which statistical tools are readily available. However, only a few methods have been proposed to focus on the direct analysis of gene expression correlation along the chromosomes. The direct analysis of correlations may have different purposes:

(i) one can aim at detecting all potential chromosomal domains of co-expression, then investigating to which extend known causal mechanisms are responsible for the observed co-expression patterns,
(ii) one can aim at detecting chromosomal domains of co-expression where correlations are not caused by already known sources of regulation, in order to identify new potential mechanisms impacting transcription.

Addressing problems (*i*) and (*ii*) is crucial to fully understand transcriptional deregulation and/or to model gene regulation. We first consider problem (*i*) and provide a precise definition of our purpose: one aims at identifying correlated regions, i.e. blocks of neighboring genes, the expression of which displays correlations across patient samples that are significantly higher than expected. Indeed, it has been observed that background correlation between adjacent genes along the genome does exist. This background correlation should not be confounded with the co-expression that can be locally observed due to the aforementioned mechanisms. Consequently, we do not consider here methods that only account for this background correlation in the statistical modeling (for instance to improve the detection of differentially expressed genes), such as [24], [40] or [30]. Also note that we focus on methods that detect correlated regions on the basis of expression data solely. This excludes strategies that look for clusters of adjacent genes based on correlations between gene expression and a given phenotype or response, such as Rendersome [24], DIGMAP [41] or REEF [10].

Several approaches have been proposed to tackle problem (*i*). CluGene [13] uses a clustering method accounting for the chromosomal organization of the genes, while G-NEST [20] and TCM [28] rely on sliding windows procedures. The principle of the latter approach is to compute correlation scores for genes falling within the window, then to detect local peaks of high correlation scores. While these procedures have been successfully applied to cancer data, all tackle the detection of correlated region using heuristics. As such, they suffer from classical limitations associated with these techniques, including local optimum (for clustering algorithms) or detection instability according to the choice of the window size (for sliding windows).

It is now well known that the problem of finding regions in a spatially ordered signal can be cast as a segmentation problem, for which standard statistical models exist, along with efficient algorithms to find the globally optimal solution [3]. According to our definition, the detection of correlated regions boils down to the block-diagonal segmentation of the correlation matrix between gene expressions. Such an approach has been proposed in image processing [22], finance [18] and bioinformatics for CNV analysis [42], but to the best of our knowledge it has never been considered for the detection of correlated expression regions.

While problem (*i*) can be addressed on the basis of only expression data, problem (*ii*) requires the additional measurement of the signal one needs to account for. For example, consider that one seeks for locally expressed co-regulation events that are not due to copy number variations but due to other causes such as epigenetic mechanisms. The strategy we adopt here consists in first correcting the expression data for potential cancer CNV contribution, then in applying the procedure described to solve problem (*i*) on the corrected signal. The corrected signal is obtained by regressing the initial expression signal on the CNV signal. Although quite simple, the strategy turns out to be efficient in practice. An alternative strategy would be to jointly model both the expression and the signals to correct for, and then propose within this framework a correction. Such a strategy would necessitate to adapt the modeling to the specific combination of signals one has at hand. In comparison, the regression procedure proposed here can be applied to any kind and any number of signals one needs to correct for.

The outline of the present article is the following. In Section ‘Correlation matrix segmentation’ (Methods) we propose a parametric statistical framework for the problem of correlated region identification. Finding regions of co-regulated genes can then be achieved by maximum likelihood inference (to find the boundaries of each region along with their correlation levels). Moreover, we propose a procedure to correct for known sources of correlation. An exact test procedure to assess the significance of the correlation with respect to background correlation is proposed in Section ‘Assessing correlation significance’ (Methods). We introduce a simple procedure to correct expression data beforehand for some known (and quantified) sources of correlation. Because the background correlation level is a priori unknown, an estimator of this quantity is also proposed. The performance of the resulting procedure, called SegCorr hereafter, is illustrated in Section ‘Simulation study’ (Results) on simulated data, along with a comparison with the TCM algorithm proposed in [28]. Finally, a case study on cancer data is presented in Section ‘Bladder cancer data’ (Results), in which we identify some regions with high correlation between gene expression and the local DNA methylation level.

## Methods

### Correlation matrix segmentation

#### Statistical model

We consider the following expression matrix:

$$ {Y} =\left[ \begin{array}{ccc} {Y}_{11} & \cdots & {Y}_{1p} \\ {Y}_{21} & \cdots & {Y}_{2p} \\ \vdots & \ddots & \vdots \\ {Y}_{n1} & \cdots & {Y}_{np} \end{array} \right] $$

where *Y*_*ij*_ stands for the expression of gene *j* (*j*=1,…,*p*) observed in patient *i* (*i*=1,…,*n*). The *i*-th row of this matrix is denoted *Y*_*i*_ and corresponds to the expression vector of all genes in patient *i*. In order to detect regions of correlated expression, we consider the following statistical model. Profiles {*Y*_*i*_}_1≤*i*≤*n*_ are supposed to be i.i.d, normalized (centered and standardized), following a Gaussian distribution with block-diagonal correlation matrix *G*:

1 $$ G =\left[ \begin{array}{ccc} \Sigma_{1} & & \\ & \Sigma_{k} & \\ & & \Sigma_{K} \\ \end{array} \right] \quad \text{with} \quad \Sigma_{k} =\left[ \begin{array}{ccc} 1 & \cdots & \rho_{k} \\ \vdots & \ddots & \vdots \\ \rho_{k} & \cdots & 1 \end{array}\right].  $$

The model states that genes are spread into *K* contiguous regions, with respective lengths *p*_*k*_ (*k*=1,…,*K*, $\sum _{1 \leq k \leq K} p_{k} = p$), the length of a region being the number of genes it contains. Genes belonging to different regions are supposed to be independent, whereas genes belonging to a same region are supposed to share the same pairwise correlation coefficient *ρ*_*k*_. This amounts to assume that some specific effect (e.g. methylation) affects the expression of all genes belonging to the region. More specifically, let *U*_*k*_ denote the vector of the region effect (accross patients). For all genes *j* from region *k*, the model can be written as *Y*_*ij*_=*U*_*ik*_+*E*_*ij*_. The error terms *E*_*ij*_ are all independent and independent from *U*_*ik*_ such that $\mathbb {V}(U_{ik})/\mathbb {V}(Y_{ij}) = \rho _{k}$, where $\mathbb {V}(U)$ stands for the variance of *U*.

While different technologies (microarrays, RNA-seq) may provide different types of signal (continuous, counts), an appropriate transformation may be applied to make the Gaussian assumption reasonable. For example, in the context of segmentation, [7] showed that Gaussian segmentation applied to log(1+*x*)-transformed RNA-seq data performs as well as negative binomial segmentation applied to the raw data.

#### Accounting for known sources of regulation

As mentioned in the Introduction, a second task (*ii*) can be to detect correlated regions which are not due to an already known mechanism. To this aim, one may first correct the expression signal using the following regression model :

2 $$ {Y}_{ij} = \beta_{0} + \beta_{1} x_{ij} + \epsilon_{ij},  $$

where *x*_*ij*_ stands for the covariate observed in patient *i* for gene *j*. For instance, in the illustration of Section ‘CNV-dependent regions’, *x*_*ij*_ is the copy number associated to patient *i* at location of gene *j*. The corrected signal is then $\widetilde { {Y} }_{ij}= {Y}_{ij}- \widehat {\beta }_{0} - \widehat {\beta }_{1} x_{ij}$. Note that $\widehat {\beta }_{0}$ and $\widehat {\beta }_{1}$ can be obtained as ordinary least-square estimates. Indeed, it suffices to assume that (*ε*_*ij*_) are independent among patients (but not among genes) to get the standard linear regression estimates (see [2], Chapter 8). Once the correction has been made, the model described in Section ‘Statistical model’ can be applied to the corrected signal $\widetilde {Y}_{ij}$.

Note that the correction procedure could be based on more sophisticated modellings of the relationship between gene expression and mechanisms such as CNV or methylation, e.g. the ones proposed in [19, 23, 38]. The difference between the observation and the prediction obtained from one such model (i.e. the residuals) could then be used as the corrected signal.

Lastly, the proposed correction procedure can be adapted straightforwardly to handle count data such as provided by RNAseq technologies. Indeed, Model (2) can be rephrased in the generalized linear model framework and Pearson residuals can be used as $\widetilde {Y}_{ij}$ (see e.g. [12] for a general introduction or [15] for the specific case of negative binomial regression).

### Inference of correlated regions

Parameter inference in Model (1) amounts to estimating the number of regions *K*, the region boundaries 0=*τ*_0_<*τ*_1_<⋯<*τ*_*K*_=*p*, and the correlation parameters *ρ*_1_,…,*ρ*_*K*_ within each of these regions. Here, we consider a maximum penalized likelihood approach. First, we show that for a given *K* the optimal region boundaries and correlation coefficients can be efficiently obtained using dynamic programming. The number of regions can then be selected using a penalized likelihood criterion. For a fixed *K*, the estimation problem can be formulated as follows:

3 $$\begin{array}{@{}rcl@{}}  \arg\max_{\tau_{1} < \dots < \tau_{K-1}} \max_{\rho_{1}, \ldots,\rho_{K}} \mathcal{L} \end{array} $$

where the log-likelihood $\mathcal {L}$ is −(*n* log|*G*|+tr[*YG* ^−1^(*Y*)^⊤^])/2. Here, thanks to the block diagonal structure of the correlation matrix in Model (1), the log-likelihood can be rewritten as

4 $$\begin{array}{@{}rcl@{}} -2 \mathcal{L} & = & \sum\limits_{k} \left\{ n\log|\Sigma_{k}| + \text{tr}\left[ {Y}^{(k)} \Sigma_{k}^{-1} ({Y}^{(k)})^{\top} \right] \right\}\\ &=& -2\sum\limits_{k} \mathcal{L}(\tau_{k-1}+1,\tau_{k}) \quad = - 2\sum\limits_{k} \mathcal{L}_{k} \end{array} $$

where *Y* ^(*k*)^ stands for the set of expression from *Y* corresponding to genes included in the *k*-th region, and $\mathcal {L}_{k}=\mathcal {L}(\tau _{k-1}+1,\tau _{k})$ is the log-likelihood corresponding to region *k*, i.e. corresponding to measurements of genes from *τ*_*k*−1_+1 to *τ*_*k*_. While log-likelihood (4) is derived in a Gaussian setting, it can be used for count data, as the Pearson residuals mentioned in Section ‘Accounting for known sources of regulation’ have an approximate Gaussian distribution.

Thanks to the additivity of the likelihood over the regions, the optimization problem (3) boils down to

5 $$\begin{array}{@{}rcl@{}}  \arg\max_{\tau_{1} < \dots < \tau_{K-1}} \sum\limits_{k} \max_{\rho_{k}} \mathcal{L}_{k}. \end{array} $$

**Inference when** ***K*** **is fixed** We first show that for a given region *k* with known boundaries, explicit expressions can be obtained for both the ML estimator $\widehat {\rho }_{k}$ and the likelihood $\mathcal {L}_{k}$ at the optimum:

#### **Lemma 1**

For a region *k* with fixed boundaries [*τ*_*k*−1_+1,*τ*_*k*_], the maximum of $\mathcal {L}_{k}$ with respect to *ρ*_*k*_ is reached for

$$\widehat{\rho}_{k} = \frac{\sum_{j=\tau_{k-1}+1}^{\tau_{k}}{\sum_{\ell=\tau_{k-1}+1}^{\tau_{k}}{\widehat{G}_{j\ell}}}-p_{k}}{p_{k}^{2} - p_{k}} $$

where $\widehat {G}_{j\ell } := n^{-1} \sum _{i=1}^{n} {Y}_{ij} {Y}_{i\ell }$. Furthermore, the maximal value of $\mathcal {L}_{k}$ is given by:

$${} -2 \widehat{\mathcal{L}}_{k}\! =\! n\left[p_{k}\! +\! (p_{k}\,-\,1)\log{\left(1\! -\! \widehat{\rho}_{k} \right)}\! +\! \log{\left(1\! +\! (p_{k}\,-\,1)\widehat{\rho}_{k} \right)}\right]. $$

The proof is given in Additional file 1. The expression of Problem (5) is now

$$\arg\max_{\tau_{1} < \dots < \tau_{K-1}} \sum\limits_{k} \widehat{\mathcal{L}}_{k} \ $$

which is additive with respect to the $\widehat {\mathcal {L}}_{k}$ terms that can be straightforwardly computed thanks to Lemma 1. Consequently, optimization can be performed via Dynamic Programming (DP, [17], [25]). The optimal boundaries, and correlation estimators can be obtained at computational cost $\mathcal {O}(Kp^{2})$.

Lasso-type approaches have been proposed to tackle segmentation problems in a faster way (see e.g. [36]). First, note that such methods rely on a relaxation of the original problem, so that the result may be different from the exact solution of problem (3). Furthermore, in the context of matrix segmentation, such approaches have been proposed ([5, 21]), which do not allow to capture the longitudinal structure (i.e. blocks of neighboring genes).

**Model selection** To choose the number of regions, we adopt the model selection strategy proposed in [17]. For each 1≤*K*≤*K*_max_, we define the maximal log-likelihood for *K* regions as

$$L_{K} = \max_{\tau_{1} < \dots < \tau_{K-1}} \sum\limits_{k} \widehat{\mathcal{L}}(\tau_{k-1}+1, \tau_{k}) \ . $$

Furthermore, the normalized log-likelihood is defined as

$$\widetilde{L}_{K} = \frac{L_{K_{\max}} - L_{K}}{L_{K_{\max}} - L_{1}}(\widetilde{K}_{\max} - \widetilde{K}_{1}) + 1, $$

where $\widetilde {K}_{j} = 5\times j + 2\times j \log {(p/j)}$ is the penalty function. [17] suggests to estimate the number of regions $\widehat {K}$ as the value of *K* such that $\widetilde {L}_{K}$ displays the largest slope change. Namely, we take

6 $$  \widehat{K} = \arg\min_{K} \left\{ (\widetilde{L}_{K} - \widetilde{L}_{K+1}) - (\widetilde{L}_{K+1} - \widetilde{L}_{K+2}) > S \right\},  $$

where the value of threshold *S* is predefined. Throughout the paper, we used *S*=0.7 as suggested in [17]. The robustness of the results with respect to other values for threshold *S* is investigated in Section ‘Simulation study’. This global approach (dynamic programming and model selection) has been applied with success for CNV detection (see [25] and [16] for a comparative study).

### Assessing correlation significance

It has been observed [9, 28, 32, 34] that background correlations may exist between adjacent genes along the genome, i.e. one expects the correlation level in any region to be positive. As a consequence, one has to check whether a given region exhibits a correlation level that is significantly higher than the background correlation level *ρ*_0_, that is observed by default.

**Test procedure** Once the correlation matrix segmentation is performed, it is possible to identify regions with high correlation levels by testing *H*_0_:*ρ*_*k*_=*ρ*_0_ vs *H*_1_:*ρ*_*k*_>*ρ*_0_. This can be done using the following test statistic for region *k*:

$$\begin{array}{@{}rcl@{}} T_{k} = \sum\limits_{i}^{n} \left(Y^{(k)}_{i\bullet} - Y^{(k)}_{\bullet\bullet}\right)^{2} \end{array} $$

where $Y^{(k)}_{i\bullet } = p_{k}^{-1} \sum _{j =\tau _{k-1}+1}^{\tau _{k}} Y_{ij}$ and $Y^{(k)}_{\bullet \bullet } = n^{-1} \sum _{i =1}^{n} Y^{(k)}_{i\bullet }$. Assuming Model (1) is true, test statistic *T*_*k*_ has distribution

$$\begin{array}{@{}rcl@{}} T_{k} \sim \lambda(p_{k},\rho_{k}) \chi^{2}_{n-1} \ \text{where } \ \lambda(p_{k},\rho_{k})=\frac{(1 + (p_{k}-1)\rho_{k})}{p_{k}} \ . \end{array} $$

Here $\chi ^{2}_{n-1}$ stands for the chi-square distribution with *n*−1 degrees of freedom. The proof is given in Additional file 1. We emphasize that this test is exact and does not rely on any resampling strategy.

Consequently, the *p*-value associated to region *k* is given by

$$\begin{array}{@{}rcl@{}} \mathbb{P}\left(\lambda(p_{k},\rho_{0})Z > T_{k}^{obs} \right), \text{where} \ \ Z\sim \chi^{2}_{n-1}. \end{array} $$

**Fig. 1:**
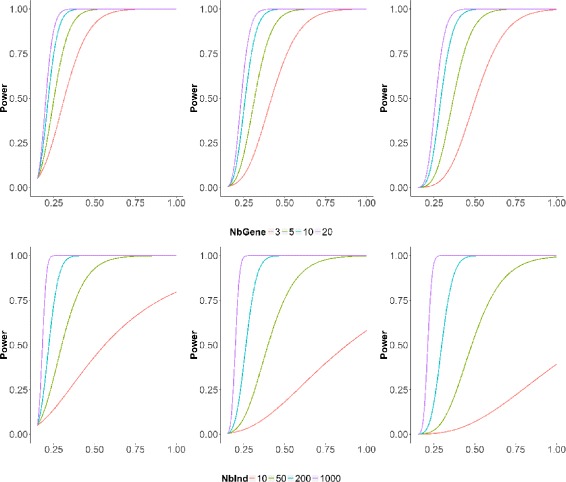
Theoretical Power. *Top*: Power curves as a function of *ρ*, for a fixed cohort size *n*=100 and varying region width *p*_0_=3,5,10,20. *Bottom*: Same graphs for a region of fixed width *p*_0_=5 but varying cohort sizes *n*=10,50,200,1000. In all graphs *ρ*_0_ is fixed at 0.15. The nominal level *α* of the test is set to 5% (*left*), 0.5% (*center*), 0.05% (*right*)

**Statistical power** We now study the ability of the proposed test to detect a region with width *p*_0_ where the correlation *ρ* is higher than in the background. The probability to detect such a region depends on both *p*_0_ and *ρ* and is given by

$$\begin{array}{@{}rcl@{}} Po(n,p_{0},\rho) &=& \Pr\{T > \lambda(p_{0}, \rho_{0})q_{n-1,1-\alpha}\}\\ &=& \Pr\left\{ Z > \frac{\lambda(p_{0}, \rho_{0})}{\lambda(p_{0}, \rho)}q_{n-1,1-\alpha}\right\} \end{array} $$

where $Z\sim \chi ^{2}_{n-1}$ and *q*_*n*−1,1−*α*_ is the 1−*α* quantile for the $\chi ^{2}_{n-1}$ distribution. Figure 1 (Top) displays the evolution of power for different values of *p*_0_ and *ρ*. Here *ρ*_0_ and *n* are fixed at 0.15 and 100, respectively. The nominal levels of *α* are 5, 0.5 and 0.05%. These levels correspond to realistic thresholds, once multiple testing corrections such as Bonferroni or FDR are performed. One can observe that even for small values of *ρ*, the power is high whatever the nominal level as long as the number of genes in the considered region is equal to or higher than 5. Figure 1 also shows that the procedure will probably fail to find regions of size 3, if the correlation is not 0.7 or higher (to obtain a power of 0.8). On the same graph (Bottom), one observes that a sample of size 50 is sufficient to efficiently detect regions of size 5, as long as the correlation is higher than 0.6. Larger samples will be required if one wants to efficiently detect regions with smaller correlation levels.

**Background correlation estimation** The test procedure requires the knowledge of parameter *ρ*_0_ that is unknown in practice. However, it can be estimated using

7 $$\begin{array}{@{}rcl@{}} \widehat{\rho_{0}}= |\underset{i>1}{\text{median}}(\text{corr}({Y}^{j-1}, {Y}^{j}))| \end{array} $$

where *Y* ^*j*^ stands for the vector of expression of gene *j* for the *n* patients. Under the assumption that most pairs of adjacent genes display a *ρ*_0_ correlation, i.e. only a few number of regions with moderate sizes exhibit a high level of correlation, $\widehat {\rho _{0}}$ is a robust estimator of the background correlation. The behavior of estimator (7) is investigated in Section ‘Simulation study’.

## Results

### Simulation study

In this section, we first study the quality of the proposed estimator of *ρ*_0_. Then we study the ability of SegCorr to detect correlated regions and compare its performance with this of TCM algorithm. The robustness of the method with respect to the choice of the model selection threshold *S* will be investigated in Section ‘Study of the model selection threshold *S*’ on real data, since very little difference were observed on the simulated data (results not shown). We also study the robustness of our procedure to a scheme where the within-region correlation is variable.

#### Simulation design

**Fig. 2:**
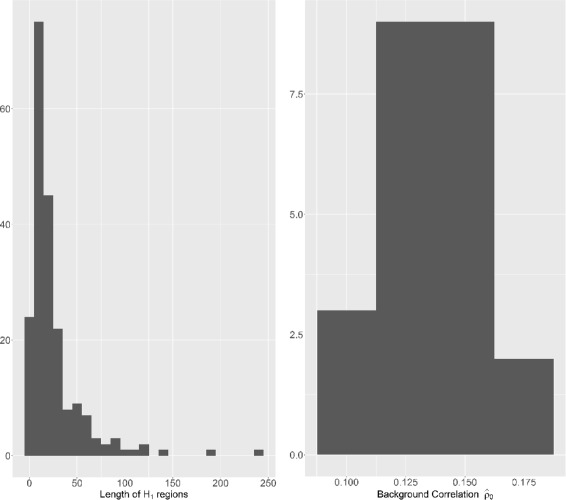
Simulation Design. *Left*: Length of *H*_1_ regions in the reference dataset. *Right*: Distribution of the background correlation $\hat {\rho }_{0}$ obtained from the reference data according to the segmentation obtained in [34]

Scenario 1 (Easy case): the regions are defined as in [16]: each patient has one chromosome containing *p*=500 genes and 4 regions with respective lengths *p*_*k*_=5,10,20,40. Three values are considered for *ρ*_0_:.08,.18,.28. These values are inspired by the distribution (displayed in Fig. 2) of *ρ*_0_ from Scenario 2. *ρ*_0_=.28 is higher than observed in [34], making the detection problem more difficult. *ρ*_1_ varies between.3 and.9. Scenario 2 **(Realistic case – constant correlation on** ***H***_**1**_ **regions):** each patient has 22 chromosomes. The length of the chromosomes, the number of regions within each chromosome and their respective sizes are the same as in the results from [34]. *ρ*_0_ is specific to each chromosome and estimated on the same dataset. *ρ*_1_ varies between.3 and.9. Scenario 3 **(Realistic case – variable correlation on** ***H***_**1**_ **regions):** the design is the same as in Scenario 2, except that *ρ*_0_ is fixed to.18. Furthermore, for each *H*_1_ region covariance matrix is drawn from a *p*_*k*_-variate Wishart distribution $\mathrm {W}_{p_{k}}\left (S,\nu \right)$ where the entries of the matrix *S* are one on the diagonal and *ρ*_1_=.5 elsewhere and *ν* is the number of degrees of freedom. Small values of *ν*, result in a higher variance, making the detection more difficult. Because *ν* has to be greater or equal to *p*_*k*_, we took *ν*=*p*_*k*_×2^*β*^, where *β*=(0.5,1,1.5,…,5). So the variability decreases as *β* increases.

For each scenario, samples of n = 50 and 100 patients were considered and, for each combination (*n*, *ρ*_0_, *ρ*_1_) the simulation was replicated 100 and 20 times, for the first and the last two scenarios respectively.

#### Quality of the *ρ*_0_ estimator

**Fig. 3:**
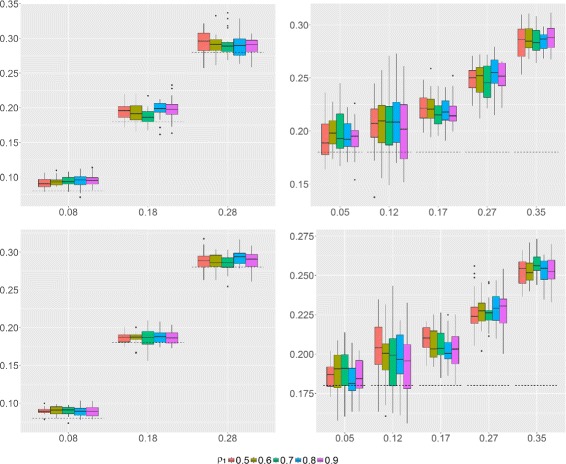
*ρ*_0_ estimator. *Left*: estimation of *ρ*_0_ for chromosome 5 under different levels of both *H*_0_ and *H*_1_ correlations (*ρ*_0_=0.08,0.18 and 0.28). *Dashed lines* indicate the true *ρ*_0_. *Right*: estimation of *ρ*_0_ for *ρ*_0_=0.18 and different levels of *H*_1_ correlations according to the fraction of *H*_1_ correlations (the results are showed for five typical chromosomes only). *Top*: *n*=50. *Bottom*: *n*=100

For this study, we consider Scenario 2. Figure 3 illustrates the estimation accuracy of *ρ*_0_ under different levels of both *H*_0_ and *H*_1_ correlations on chromosome 5. Estimator (7) yields over-estimated values of the true background correlation level. One observes that the overestimation does not depend on the correlation level in *H*_1_ regions, thanks to the use of the median. Still, as expected, it is linked to the proportion of pairs of adjacent genes with *H*_1_ correlations, as showed in Fig. 3. Importantly, while over-estimation of *ρ*_0_ will result in a decrease of power, it will not increase the false positive rate (FDR or FWER).

#### Performance evaluation

To assess the performance of SegCorr, the true positive rate (TPR = sensitivity), false positive rate (FPR =1− specificity) and area under the ROC curve (AUC) were considered. These criteria were first computed at the gene level. However, as the goal is to identify correlated regions, a definition of TPR and FPR at the region level was adopted. We considered the intersection between the true and the estimated segmentations and computed the number of true/false positive/negative regions. This amounts at classifying each gene into one of four status (true/false × positive/negative) and then to merge neighboring genes sharing a same status into regions. The status of a region is given by the status of its genes. Consequently, criteria computed at the region level are more stringent as they measure the precision of region boundary estimation.

**Fig. 4:**
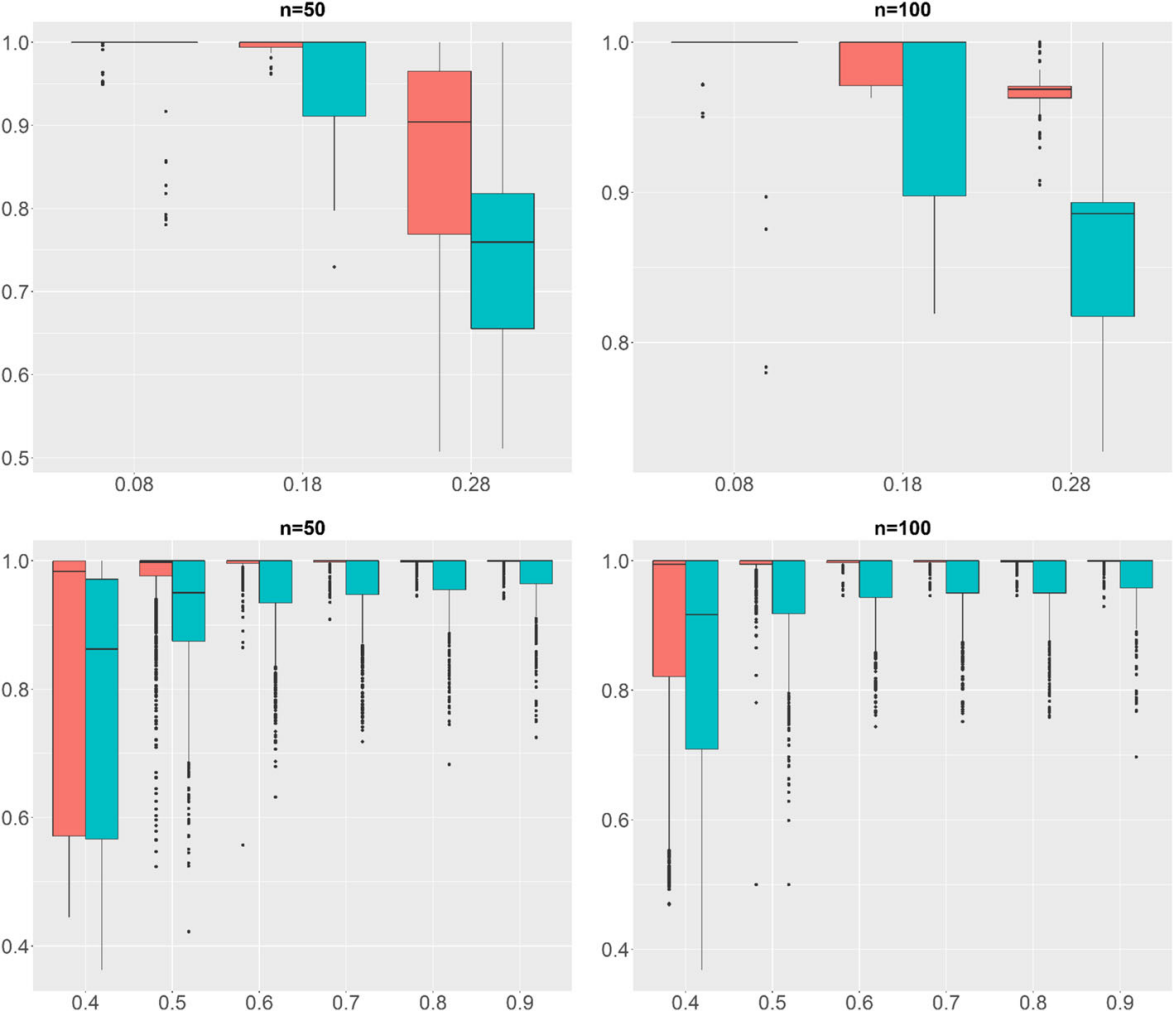
AUC for Simulation Design 1 and 2. AUC at the gene level (*red*) and region level (*blue*). The higher the AUC the better. *Top*: Simulation design 1 with fixed *ρ*_1_=.5 (*x*-axis: *ρ*_0_). *Bottom*: Simulation design 2 (*x*-axis: *ρ*_1_)

Figure 4 (top) shows the AUC for Scenario 1 under various configurations, with *ρ*_1_ fixed at 0.5. When *ρ*_0_ is between 0.08 and 0.18, most regions are correctly detected. For *ρ*_0_=0.28 (a value higher than what is observed on the reference dataset, see Fig. 2), the task becomes difficult and the performance deteriorates.

For Scenario 2, the behavior of SegCorr was explored under different *ρ*_1_. Obviously the task becomes easier when *ρ*_1_ gets larger. Figure 4 shows that SegCorr performs well when 0.5≤*ρ*_1_≤0.9. When *ρ*_1_≤0.5, (remind that the background correlation can be as high as 0.2, see Fig. 2) although the performances remain good at the gene level, the boundaries of the regions are detected less accurately.

#### Comparison with the TCM algorithm

**Fig. 5:**
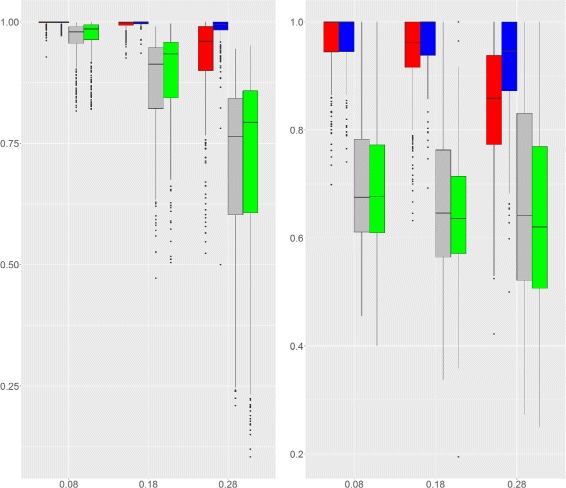
AUC for SegCorr and TCM (Scenario 2). AUC of the SegCorr (*n*=50-*red*, *n*=100-*blue*) and TCM (*n*=50-*grey*, *n*=100-*green*) algorithms for Scenario 2 as a function of *ρ*_0_. *Left*: gene level. *Right*: region level

SegCorr was compared with the TCM algorithm introduced by [28] for the detection of regional correlations. The choice of the TCM as a competing method was based on the availability of the code. Indeed, the code of CluGene [13] is not currently available and this of G-NEST [20] relies on obsolete linux packages. Figure 5 displays the AUC achieved by SegCorr and TCM under Scenario 2 for *ρ*_1_=0.5. When *ρ*_0_ is large (*ρ*_0_=0.28), one observes that the mean performance of both methods are comparable with higher variability for SegCorr at the gene level and at the region level for TCM. Since the aim is to detect regions rather than genes, the SegCorr procedure seems more appropriate. For small or medium values of background correlations (*ρ*_0_=0.08,0.18) SegCorr achieves better AUC than TCM at both the gene and the region levels. As a conclusion, SegCorr appears to be a more consistent and efficient procedure to detect correlated regions. Similar performance between SegCorr and TCM can be observed for other values of *ρ*_1_, results not included here.

**Fig. 6:**
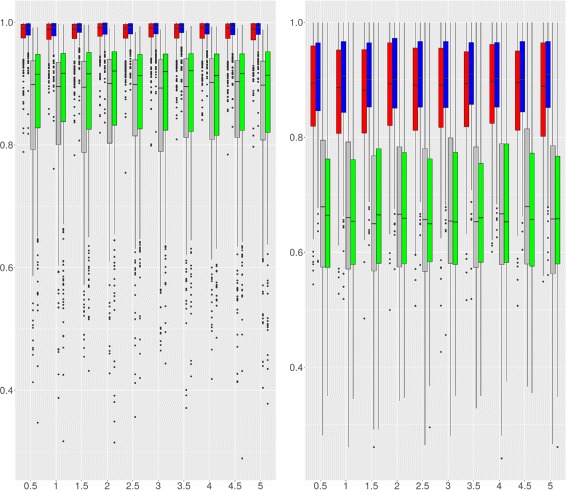
AUC for SegCorr and TCM (Scenario 3). AUC of the SegCorr (*n*=50-*red*, *n*=100-*blue*) and TCM (*n*=50-*grey*, *n*=100-*green*) algorithms for Scenario 3 as a function of *β*. *Left*: gene level. *Right*: region level

Figure 6 illustrates the performance of SegCorr and TCM under Scenario 3. As in the previous case, SegCorr outperforms TCM both on the gene and region level.

We observe that the performance of both algorithms remains unchanged between the different values of *β*. Further investigations (results not shown) show that classification errors predominantly occur in small regions with or without variability. The simulation shows that only the mean correlation within the blocks matters and that the proposed method is robust to intra-region variability of correlations.

On an Intel i7-4790 CPU processor at 3.60GHz, the CPU times is 74s for SegCorr and 61s for TCM for the bladder cancer dataset. However, in practice TCM must be executed many times in order to manually tune its input parameters (such as the window size and the threshold). On the contrary, SegCorr has to be run only once.

### Bladder cancer data

In this section, we apply SegCorr on a bladder cancer dataset described in Section ‘Data presentation’ below. It is now well known that copy number variation (CNV) impacts gene expression [29]. Here our goal is to detect regions where the correlation is not due to CNV occuring in cancer. Therefore we correct the expression signal for CNV variation according to the strategy described in Sections ‘Accounting for known sources of regulation’ and ‘Procedure for CNV correction’. The effect of this correction is investigated in Section ‘CNV-dependent regions’. Lastly, Section ‘CNV-independent regions’ illustrates the biological results obtained after correction for CNV.

#### Data presentation

The dataset consists of *n*=403 bladder tumors. Gene expression have been measured using RNA-seq. The number of genes per chromosome ranges from 293 to 1695 (with average 702). Additionally CNV data have been obtained with Affymetrix Genome wide SNP 6.0 arrays and methylation data with Illumina Human methylation 450k arrays. All RNA-seq, SNP and methylation data were dowloaded from the TCGA open-access HTTP directory (https://portal.gdc.cancer.gov/projects/TCGA-BLCA) and are level 3 data.

#### Study of the model selection threshold *S*

**Fig. 7:**
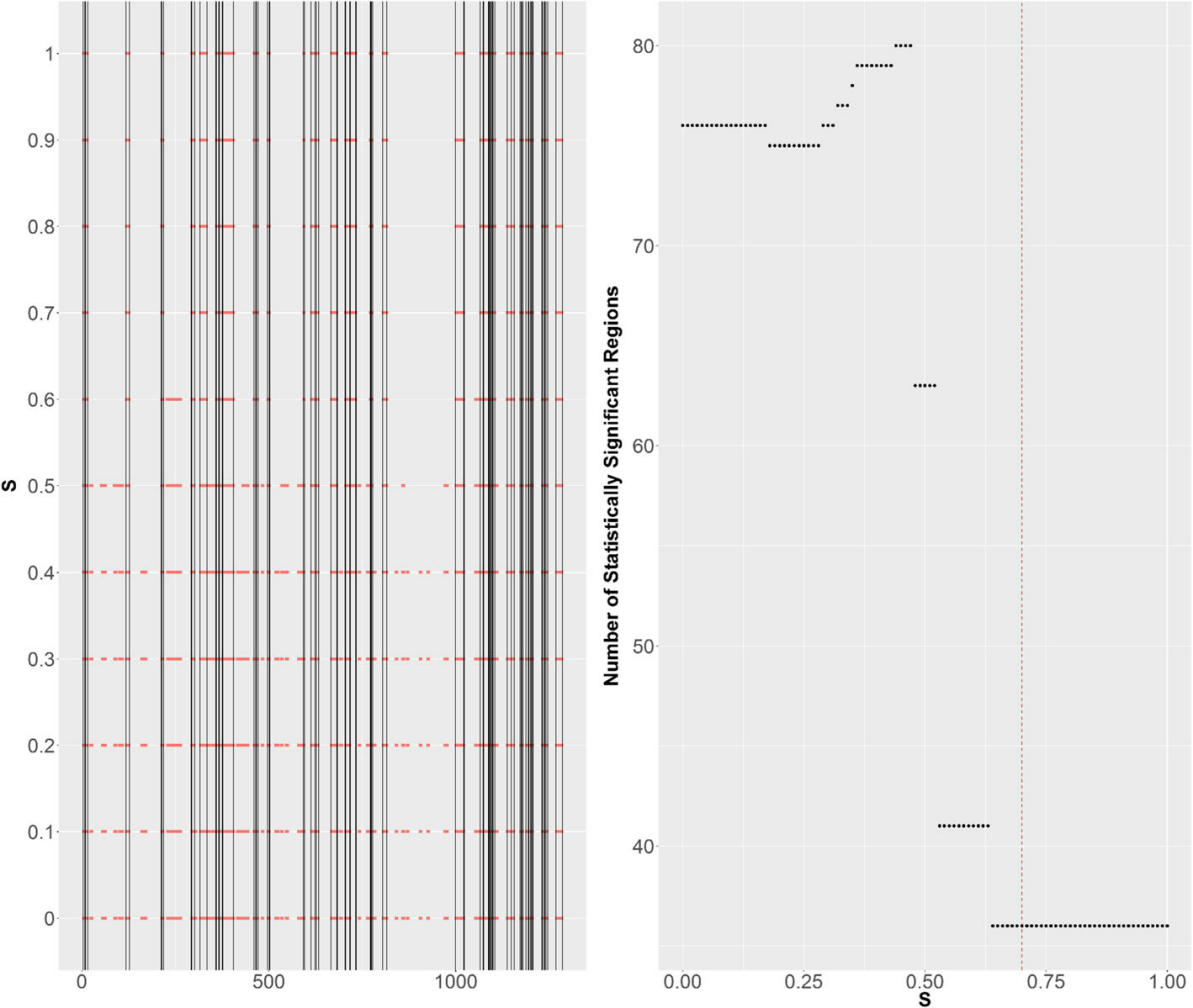
Choice of *S*. *Left*: statistically significant regions in *red* obtained for different values of *S*. The *vertical lines* correspond to the ones obtained with the default value of *S* we considered (*S*=0.7). *Right*: number of statistically significant regions for different values of *S*. The *dotted vertical red line* corresponds to *S*=0.7

For the model selection criterion, the threshold *S* (defined in Section ‘Inference of correlated regions’, Eq. (6)) must be tuned in such a way to avoid under/over-segmentation. The smaller the value of *S* the higher the number of segments. As stated in Section ‘Inference of correlated regions’, *S* was fixed to 0.7 as advocated in [17]. Figure 7 shows the evolution of the number and location of *H*_1_ regions detected by SegCorr according to *S* on a typical chromosome (chromosome 3). One can see that most of these *H*_1_ regions are stable for values of *S* between 0.6 and 0.9. Still, the value of *S* may need to be adapted when applied to other data-type or to another dataset. The choice of *S* can be parametrized in the SegCorr R package, with default value 0.7.

#### Procedure for CNV correction

To correct the expression signal from CNV, one first needs to detect the CNV regions from the SNP array signal. To this aim, we consider the segmentation method proposed by [26] implemented in the R package cghseg. Denote *SNP*_*it*_ the SNP signal of patient *i* at position *t*, the model writes

8 $$\begin{array}{@{}rcl@{}}  SNP_{it}=\mu_{ik}+E_{it}\ \ \text{if} t \in I_{k}^{i}=\,\left[t_{k-1}^{i}+1,t_{k}^{i}\right]. \end{array} $$

where the *E*_*it*_ are i.i.d centered Gaussian with variance *σ* ^2^. The method estimates the number of regions, the boundaries of the regions, denoted $\hat {t}_{k}^{i}$ and the signal mean within each region *k* in patient *i*, denoted $\hat {\mu }_{ik}$. This procedure may be adapted to count data such as provided by DNAseq data, for which dedicated segmentation tools exist (see e.g. [8]).

We then use the regression model (2) to make the correction where *x*_*ij*_ is the mean $\hat {\mu }_{ik}$ obtained previously if the SNP position *t* corresponds to gene *j* of the expression signal in patient *i*. The TCGA expression data arise from RNAseq but are provided as read counts or normalized read counts (RSEM). Then the dataset was normalized using the log(*x*+1) method as provided in https://genome-cancer.ucsc.edu/. Finally, we directly applied Model (2) to the normalized RNAseq data.

Still, as often in RNAseq, an important proportion of zero is observed. Genes with null expression in all samples were removed. For the remaining zeros, we either left them when fitting the regression model, or removed them and then set the corresponding residual $\widetilde {Y}_{ij}$ to 0 (note that, in the last option, these observations do not contribute to the estimation of the between-gene correlation, as the mean of the residuals is 0 by construction). Both options were found to provide similar results, so only the ones obtained with the first option are displayed in the following.

Since the SNP and expression signals are not aligned, there might be either one, many or no SNP probes that belong to the corresponding gene region. We then propose to define *x*_*ij*_ as follows : if one or many probes are related to gene *j*, mean $\hat {\mu }_{ik}$ or the average of the different means is considered respectively; if there is no probe, a linear interpolation is performed.

#### CNV-dependent regions

**Fig. 8:**
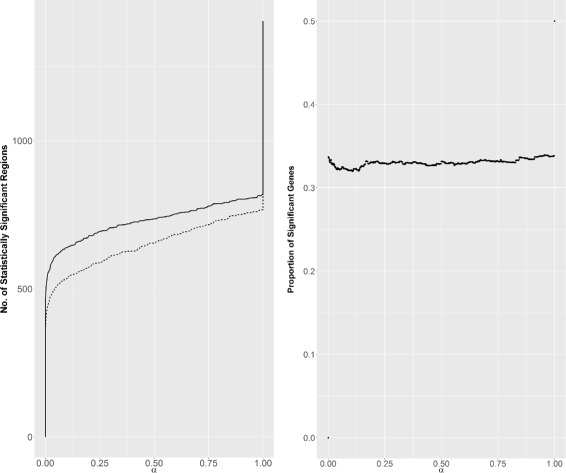
Bladder Regions. *Left*: Number of statistically significant regions as a function of *α* (*solid line*: corrected signal, *dotted line*: raw signal). *Right*: proportion of significant genes common in the two signal as a function of *α*

We first investigate the effect of CNV correction (described in Section ‘Procedure for CNV correction’) by comparing the results obtained on the raw and corrected signals. Figure 8 displays the number of significant *H*_1_ regions as a function of the test level *α* for both the raw and corrected signals. For small values of *α* (which are typically used for testing significance), the number of detected regions are quite similar. However, only one third of the detected genes are common, meaning that the regions detected with the two signals are quite different. Furthermore, as the correction removes all effects due to CNV, the estimated background correlation is lower in the corrected signal than in the raw signal (mean decrease across all chromosomes of.07). This makes the test we propose more powerful and explains why, while CNV-due regions are removed, the number of detected regions for a given *α* remains about the same.

To illustrate this phenomenon more precisely, we considered a set of four regions in chromosomes 3, 8, 10 and 12 known to be associated with CNV in bladder cancer [31, 35, 39]. These regions, given in Table 1, are detected by SegCorr when applied to the raw expression data. When considering the corrected signal, these regions are not detected any more. For the region in chromosome 10, the background correlation was $\widehat {\rho }_{0} = 0.221$ and the correlation within this region was $\widehat {\rho }_{k} = 0.405$, resulting in a highly significant *p*-value: 8.25e-06. After correction we get $\widehat {\rho }_{0} = 0.152$ and $\widehat {\rho }_{k} = 0.134$, which results in a non-significant *p*-value: 0.623.

**Table 1:** Four examples of CNV-dependent regions

Chrom.	Genes
3	*TSEN2, MKRN2, RAF1*
8	*ZNF703, ERLIN2, PROSC, GPR124, BRF2, RAB11, FIP1, ADRB3, EIF4EBP1, ASH2L, STAR, LSM1, BAG4, DDHD2, PPAPDC1B, WHSC1L1, LETM2, FGFR1, TACC1, PLEKHA2, HTRA4, TM2D2, ADAM9*
10	*ASB13, GDI2, ANKRD16, FBXO18*
12	*MDM1, RAP1B, NUP107, SLC35E3, MDM2, CPM, CPSF6, LYZ, YEATS4, FRS2, CCT2, BEST3, RAB3IP, CNOT2*

More generally, over the 119 regions solely detected on the raw signal with p-value smaller than 5% (before multiple testing correction), one third (44) get non significant when considering the corrected signal. This explains a substantial part of the difference between the regions detected on raw and corrected signals. This also shows that the proposed CNV correction strategy performs reasonably well.

#### CNV-independent regions

**General description** When applied to the CNV-corrected expression signal, SegCorr detected 588 significant regions (adjusted *p*-value ≤0.05) which are distributed throughout the genome (an average of 25 regions per chromosome). Among these regions, 135 regions contained well known gene family clusters such as the HOXA, HOXB, HOXD clusters, several KRT clusters, the epidermal differentiation complex, and HLA gene families clusters whose expression is known to be co-regulated [33]. We next undertook a Gene Ontology terms analysis with genes contained in the significant regions and identified an enrichment of genes belonging to the keratinization pathway (p-value 4.09E-19 and FDR q-value 9.01E-16). The expression of this pathway is strongly associated with a subgroup of bladder cancer called basal-like bladder cancer [27].

**Epigenetic regions** Apart for CNV, DNA methylation is one of the possible explanations for expression correlation. We first investigated whether the correlation between gene expression and DNA methylation is higher in significant regions when CNV correction is applied. The mean correlation varies marginally when considering the significant regions altogether. This suggests either that methylation is not a systematic cause of expression correlation or that the available signal is too noisy to detect methylation effect.

Still SegCorr allowed us to detect regions where DNA methylation is associated with expression correlation. More specifically, we now present one such region where the observed correlation is not due to CNV and can be associated with an epigenetic mark. This region located on chromosome 17 contains seven genes (*HOXB2, HOXB3, HOXB4, HOXB5, HOXB6, HOXB7, HOXB8*: $\widehat {\rho }_{k}= 0.717$, *p*-value = 7.94e-62). Three genes from this regions have already been studied by [37] and has been referred to as 17-7.

**Fig. 9:**
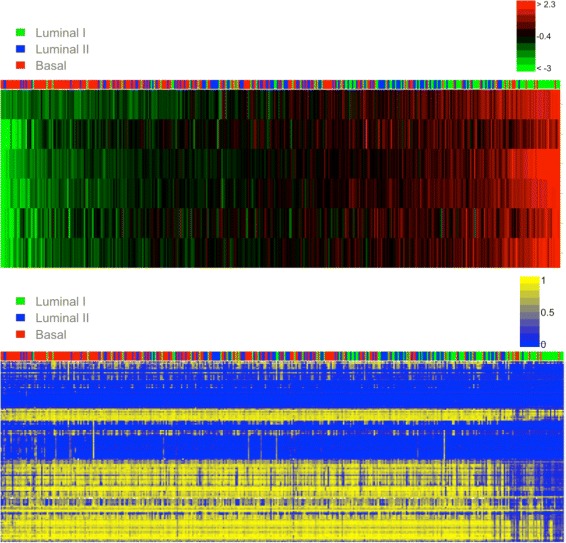
Heatmaps for Region with Epigenetic Mark. Expression (*top*) and methylation (*bottom*) data from Region 17-7. The tumors have been ordered according to the average expression of the genes from region 17-7. The same ordering of the tumors (*x*-axis) was kept in the *bottom* plot

Figure 9 (top) shows a clear pattern detected in both the expression data and the DNA methylation data. When classifying the patients into three groups, the right-most group displays an over-expression of the genes and a low methylation signal. The methylation of the DNA is one of several epigenetic mechanisms used by the cell to silence the expression of a gene. The tumors that expressed the HOXB gene family present an hypomethylation of the DNA and the tumors which did not express these genes have an hyper methylation of the DNA. This suggests that this region is silenced by an epigenetic mechanism associated with DNA methylation.

## Discussion

The identification of co-regulated chromosomal regions is important to fully understand the gene transcription network and to identify new mechanisms of gene regulation and their deregulations in pathological states such as cancer. In this paper, we developed a method to identify these regions and we applied it to cancer data. The method relies on a formal definition of what correlated regions are. It takes advantage of an efficient inference algorithm and a statistical testing procedure, which are both exact. We also proposed a correction strategy that allows one to investigate the possible causes of the observed correlations.

Using this method, we could identify copy number dependent and copy number independent correlated regions of expression. Copy number dependent regions correspond to genomic alterations; copy number independent regions could be due to different mechanisms, including epigenetic mechanism. We showed, for one region, which is part of the HOXB complex, that there is negative correlation between expression and DNA methylation. The detected regions should be further investigated to better understand the underlying mechanism. While the expression data used here were acquired using the RNA-seq technology, any other technology, including microarray technologies can be used as well.

In our analysis, we have assumed stretches of correlated contiguous neighboring genes. This is obviously a simplification. Within a correlated region, a gene (or a few genes) could exhibit a weak or even a negative correlation with the other genes. This could occur for different reasons: the gene can be not expressed; alternatively, the gene could be non affected by the regulation process that impacts the other ones; finally, the gene could be impacted in a opposite way compared with the other ones. Note that genes that exhibit no expression or no variation in the dataset can be detected and could be discarded before applying the analysis. While this preprocessing was not required in the present study, running the analysis without removing non-expressed genes would lower the performance of any method aimed at finding correlated (and reasonably homogeneous) regions. Alternatively, accounting for a variable number of uncorrelated genes in correlated regions is an obvious follow-up of the present work.

The proposed correction strategy could easily be generalized to more than one signal to correct for, as it does not rely on a joint modeling of all types of data at hand. Furthermore the segmentation used in the correction step enables one to deal with signals with different probe densities. Finally, this correction approach allowed us to keep all tumors in the study, as opposed to [34] were tumors with CNV in a given region were excluded when analysing this region.

Also, prior information on genes or regions could be accounted for in the segmentation step. Indeed, the likelihood $\widehat {\mathcal {L}}(\tau, \tau ')$ associated with a given region can be reweighted or penalized, the dynamic programming algorithm then applies with the same computational complexity.

## Conclusions

SegCorr is a novel statistical procedure build for the identification of adjacent co-expressed genes. Some of these regions could be attributed to copy number variation events. To this end, we propose a model to correct gene expression for CNV. This method can be extended for the correction of other data types. R package SegCorr is available on the CRAN.

## Additional file

Additional file 1 Appendix file containing a table with all the competing methods, the proof of Lemma 1 and the distribution of the test statistic. (PDF 106 kb)

## Abbreviations

AUC: Area under the curve
CNV: Copy number variation
FDR: False discovery rate
FPR: False positive rate
FWER: Family wise error rate
ROC: Receiver operating characteristic
TPR: True positive rate
TF: Transcription factors
