# Supplementary material for: SegCorr a statistical procedure for the detection of genomic regions of correlated expression
Source: BMC Bioinformatics. 2017 Jul 11;18:333. doi: 10.1186/s12859-017-1742-5 (PMC5504623; doi:10.1186/s12859-017-1742-5)
Supplement: Additional file 1 — Appendix file containing a table with all the competing methods, the proof of Lemma 1 and the distribution of the test statistic. (PDF 106 kb) [file 12859_2017_1742_MOESM1_ESM.pdf]

Other methods

Method	Requires exogeneous data	Available / maintained	Optimization algorithm
Rendersome	yes	–	–
DIGMAP	yes	–	–
REEF	yes	–	–
CluGene	no	no	heuristic
G-NEST	no	no	heuristic
TCM	no	yes	heuristic
SegCorr	no	yes	exact

Table 1: Comparison of several methods accounting for local correlation of gene expression. The last two columns are not filled for the first 3 methods (Rendersome, DIGMAP, REEF) because they pursue a purpose different from the one of SegCorr.

Proof of Lemma 1

Throughout this proof, we drop index k for sake of clarity. For a region with length p_0, the covariance matrix Σ in Equation (1) can be rewritten as:

$$\Sigma = (1-\rho)I + \rho J$$

where I stand for the $p_0 \times p_0$ identity matrix and J for the $p_0 \times p_0$ matrix with all entries equal to one. The inverse of this matrix has the form $\Sigma^{-1} = aI + bJ$ where

$$a = \frac{1}{1-\rho}, \qquad b = -\frac{\rho}{(1-\rho)\left[1-\rho+p_0\rho\right]}.$$

The determinant of Σ is

$$|\Sigma| = (1-\rho)^{p_0-1}(1-\rho+p_0\rho)$$

and the trace term $\mathrm{tr}\left[Y\Sigma^{-1}(Y)^\top\right]$ in Equation (4) yields

$$\mathrm{tr}\left(Y(aI+bJ)Y^\top\right) = anp_0 + bn\sum_{j=1}^{p_0}\sum_{k=1}^{p_0}\hat{G}_{jk}.$$

Combining all the above gives the log-likelihood for this region:

$$\begin{aligned} -2\log\mathcal{L} &= n\left[\log\left(1-\rho+p_0\rho\right) + (p_0-1)\log\left(1-\rho\right)\right] \\ &\quad + \frac{np_0}{1-\rho} - \frac{\rho n \sum_j^{p_0}\sum^{p_0}\hat{G}_{jk}}{(1-\rho)\left[1-\rho+p_0\rho\right]}. \end{aligned}$$

Optimizing this function wrt ρ gives the formula of the MLE (7). Pluging this estimate into the same function gives the contrast function given in (8).

Distribution of the test statistic

Note $Y_i^{(k)} = (Y_{i,\tau_{k-1}+1}, ..., Y_{i,\tau_k})^T$. Using the same notations as in Section 3, one has

$$Y_i^{(k)} \sim \mathcal{N}(0, \Sigma_k) \quad \Rightarrow \quad Y_{i\bullet}^{(k)} \sim \mathcal{N}\left(0, \frac{1 + (p_k - 1)\rho_k}{p_k}\right)$$

Because variables $Y_i^{(k)}$, $i = 1, ..., n$ are i.i.d so do variables $Y_{i\bullet}^{(k)}$, and consequently

$$\sum_i^n \left(Y_{i\bullet}^{(k)} - Y_{\bullet\bullet}^{(k)}\right)^2 \sim \frac{1 + (p_k - 1)\rho_k}{p_k}\chi^2_{n-1}.$$
